# Supplementary material for: Patterns of Intron Gain and Loss in Fungi
Source: PLoS Biol. 2004 Nov 30;2(12):e422. doi: 10.1371/journal.pbio.0020422 (PMC532390; doi:10.1371/journal.pbio.0020422)
Supplement: Table S1 — Also available at http://genes.mit.edu/NielsenEtAl/. (4.3 MB ZIP). [file pbio.0020422.st001.zip › NielsenEtAl/html/560.html]

AN3823.1.NCU08627.1.MG06837.1.FG00798.1


```
 CLUSTAL W (1.82) Multiple Sequence Alignments - Introns Inserted


Sequence 1: NCU08627.1	87 aa
Sequence 2: FG00798.1	87 aa
Sequence 3: MG06837.1	87 aa
Sequence 4: AN3823.1	88 aa
Alignment Length: 89 aa
Number Identitical Residues: 59 aa
Alignment Score (without introns) 2583


MG06837.1 	MENDKGEIVDL2YVPRKCSATNRIIKAQDHASVQISIAKVDENGRAVQGENHVYALCGFV
NCU08627.1	MENDRGEIVDL2YVPRKCSATGRIIKSKDHGSCQITIAKVDENGRAIQGENIIYALSGFV
FG00798.1 	MENDRGEIVDL2YVPRKCSATNRIIKAKDHGSVQISIAKVDENGRAIQGENHVYALCGFV
AN3823.1  	MENEKGEIVDL2YVPRKCSATNRIIKANDHASVQISIGKVDENGRYT-GENQTYALCGFI
          	***::****** *********.****::**.* **:*.*******   ***  ***.**:

MG06837.1 	RAMGEGDDALNRLTQRDGLLKNVFSAVR--
NCU08627.1	RAMGESDDSLNRLAQRDGLLKAVWNPQR--
FG00798.1 	RAMGESDDSLNRLAQRDGLLKSVWSGQR--
AN3823.1  	RARGESDDSLNRLTQRDGYIRNVWTASRQR
          	** **.**:****:**** :: *:.  *.
```
